# Adult woman presenting progressive enlargement of mass on the left leg

**DOI:** 10.1016/j.jdcr.2024.07.015

**Published:** 2024-08-05

**Authors:** Yilong Guo, Ning Ma, Sen Chen, Yangqun Li, Zhe Yang

**Affiliations:** a Department of Comprehensive Plastic Surgery, Plastic Surgery Hospital, Chinese Academy of Medical Sciences and Peking Union Medical College, Beijing, China; b Department of Hypospadias Plastic Surgery, Plastic Surgery Hospital, Chinese Academy of Medical Sciences and Peking Union Medical College, Beijing, China

**Keywords:** dermatological surgery, neurofibromatosis type 1, plexiform neurofibromas

## History

**Fig 1:**
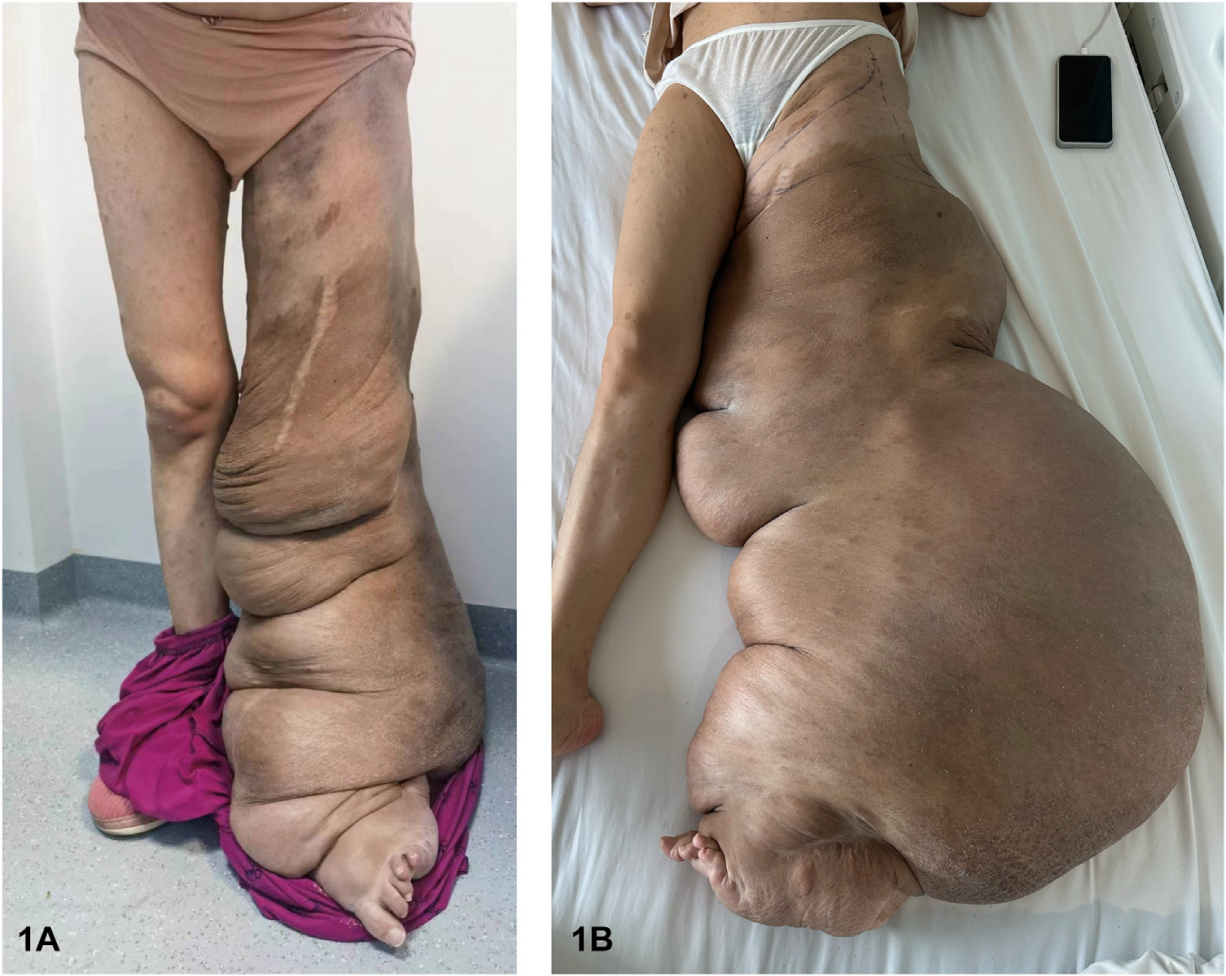

**Fig 2:**
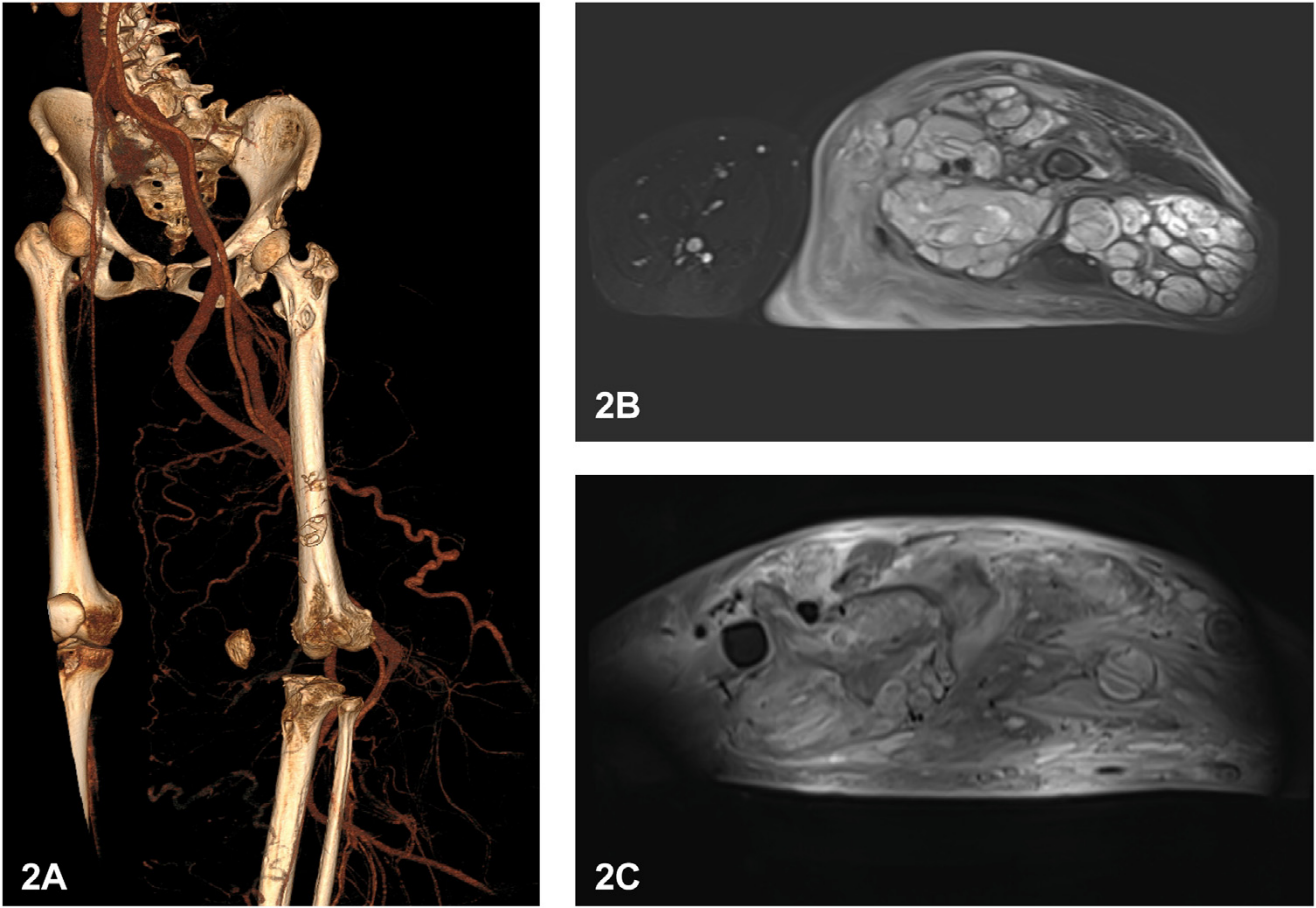

**Fig 3:**
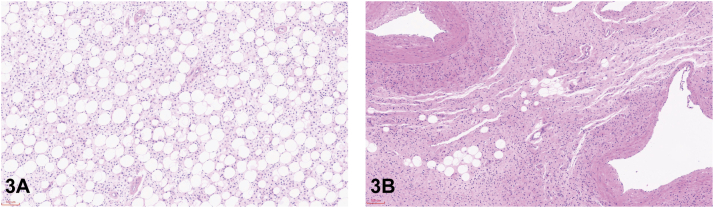

A 29-year-old woman presented with a continuously growing mass on her left leg since birth. Multiple solid, palpable, well-demarcated masses result in overlying redundant skin folds, most obvious in a dependent position (Fig 1). Computed tomography angiography revealed multiple mesh-like vascular malformations within the tumor (Fig 2). The magnetic resonance imaging (MRI) at the upper 1/3 femoral shaft level revealed multiple hyperintense lesions within the intermuscular space. The mass extends distally with increased heterogeneity and expanded extent of involvement (Fig 2). Surgical histopathology of the tumor showed the composition of spindled and round cells with myxoid stroma (Fig 3).

**Question 1: What is the most likely diagnosis?**

A. Lymphedema
B. Chronic venous disease (CVD)
C. Plexiform neurofibromas (PNs)
D. Klippel-Trenaunay syndrome (KTS)
E. Lipedema

**Answers:**

A. Lymphedema – Incorrect. Lower extremity lymphedema is caused by the excessive retention of lymphatic fluid in the interstitial compartment. The initially affected area of lower extremity lymphedema was the distal part of the leg such as the ankle, foot, and toes. The foot of the patient is completely spared.
B. Chronic venous disease (CVD) – Incorrect. CVD is associated with telangiectasias, varicose veins, swelling, and skin changes. Leg swelling of CVD often occurs in ankles and calves, while the feet are usually spared, and it can be effectively improved by leg elevation.
C. Plexiform neurofibromas (PNs) – Correct. PNs are benign nerve sheath tumors that occur commonly in individuals with neurofibromatosis type 1 (NF1). The family history of NF1 is significant in her father and aunt. The MRI result showed a well-demarcated, encapsulated, ≥3 cm lesion matching the imaging features of PNs. Histologically, they exhibit a typical composition of spindled with wavy, tapered, and comma-shaped nuclei.
D. Klippel-Trenaunay syndrome (KTS) – Incorrect. KTS is a congenital disorder characterized by capillary, lymphatic, and venous system malformation, as well as bone and/or soft tissue hypertrophy. MRI can identify the asymmetric soft tissue hypertrophy and osteohypertrophy of the leg. Leg function impairment secondary to hypertrophy can be observed in KTS, but many patients demonstrated a reduced body area rather than increased growth. By comparison, most PNs are characterized by continuous growth and PN shrinkage mainly occurs in adults. Vascular masses, dilated superficial veins, and flat hemangiomas can also be assessed by color duplex ultrasound and venography. These vascular anomalies are significant clinical features of KTS but are rarely present in PNs.^1^
E. Lipedema – Incorrect. Lipedema is associated with abnormal deposition of subcutaneous adipose, leading to the disproportional increase of lower extremities in the volume. Digital pressure on the affected limbs can induce leg pain. Leg swelling worsens with standing and cannot be effectively alleviated by limb elevation therapy.

**Question 2: Which of the following is not an urgent condition for treatment?**

A. Deteriorated patients' psychosocial status and quality of life (QOL)
B. Risk of malignant peripheral nerve sheath tumor (MPNST) transformation
C. Intolerable leg pain
D. Cafe´ au lait macules (CALMs) on her body
E. Leg function impairment

**Answers:**

A. Deteriorated patients’ psychosocial status and quality of life (QOL) – Incorrect. NF1-PN is associated with anxiety, depression, and social withdrawal. Our patient has been unable to walk or care for herself for at least 4 years. The increasing psychosocial burden and decline in QOL prompted her to seek effective treatment.
B. Risk of malignant peripheral nerve sheath tumor (MPNST) transformation – Incorrect. MPNST is a type of soft tissue sarcoma developed in NF1 patients, typically associated with progressive pain, tenderness, and paresthesia. MPNST is commonly located in the spinal root, extremities, trunk, head, and neck.^2^ MPNST carries a significant risk of recurrent and metastasis, but the symptoms are otherwise nonspecific and site-dependent. The pathological diagnostic criteria of MPNST are perivascular hypercellularity, fascicular growth, uniform spindle cells with hyperchromatic nuclei, high mitoses, and true tumor necrosis.^3^ The symptoms of stable but noticeable leg pain, continuous growth, and tenderness raised the suspicion of MPNST, prompting timely diagnosis and treatment.
C. Intolerable leg pain – Incorrect. Pain is the most documented symptom in both children and adolescents. The higher rating of pain management is associated with disease severity in youth and lower QOL in adolescents.
D. Café au lait macules (CALMs) on her body – Correct. CALMs have no tendency for malignancy. Surgical removal and laser therapy of CALMs can yield satisfying results.
E. Leg function impairment – Incorrect. Larger tumor volume is associated with the severity of symptoms, compressive signs, and motor dysfunction. MRI indicated impaired muscle structure and function. Knee and ankle joint impairment, along with the inability to walk, are pressing conditions that require timely intervention.

**Question 3: What are the best next steps in terms of treatment?**

A. Endovascular embolization
B. Debulking surgery
C. Medical treatment
D. Radiation therapy (RT)
E. Above-knee amputation surgery

**Answers:**

A. Endovascular embolization – Incorrect. Computed tomography angiography revealed that the PN is nourished by the branches of 2 major arteries. The embolization of major arteries can cause extensive tissue necrosis of the leg, and the embolization of artery branches might be ineffective in relieving the PN-related morbidities.^4^
B. Debulking surgery – Incorrect. The MRI showed extensive soft tissue involvement, and debulking surgery can barely restore the leg function when the muscle and knee joint are severely deteriorated. The residual PN is still associated with the risk of malignancy and continuous growth.^5^
C. Medical treatment – Incorrect. For such a giant PN, medical treatment can barely meet the urgency of restoring the function and improving QOL.
D. Radiation therapy (RT) – Incorrect. RT is suitable for confined benign lesions with distinctive borders. The PN in our patient is extensively involving the entire leg; the improper RT can be either ineffective or damaging.
E. Above-knee amputation surgery – Correct. The amputation at the above-knee level can resect most of the PNs in our patient and provide us with more space to safely ligate large blood vessels. Debulking surgery can also be performed simultaneously on the leg to reduce the PN volume and provide more soft tissue to achieve the conical form of the stump, thereby minimizing the difficulties in fitting the stump in the socket. Following graduated rehabilitation, the patient’s function and QOL can be significantly improved.

## Conflicts of interest

None disclosed.

## References

[1] Abdel Razek A.A.K. (2019). Imaging findings of Klippel-Trenaunay syndrome. J Comput Assist Tomogr.

[2] Carli M., Ferrari A., Mattke A. (2005). Pediatric malignant peripheral nerve sheath tumor: the Italian and German soft tissue sarcoma cooperative group. J Clin Oncol.

[3] Meyer A. (2020). Review and update in the diagnosis of peripheral nerve sheath tumors. Curr Opin Neurol.

[4] Tovo Filho R., Carnevale F.C., Curi T.Z. (2020). Surgery combined with embolization in the treatment of plexiform neurofibroma: case report and literature review. JAAD Case Rep.

[5] Fisher M.J., Blakeley J.O., Weiss B.D. (2022). Management of neurofibromatosis type 1-associated plexiform neurofibromas. Neuro Oncol.

